# Efficacy and cost-effectiveness of an outcall program to reduce carer burden and depression among carers of cancer patients [PROTECT]: rationale and design of a randomized controlled trial

**DOI:** 10.1186/1472-6963-14-5

**Published:** 2014-01-06

**Authors:** Patricia M Livingston, Richard H Osborne, Mari Botti, Cathy Mihalopoulos, Sean McGuigan, Leila Heckel, Kate Gunn, Jacquie Chirgwin, David M Ashley, Melinda Williams

**Affiliations:** 1 Faculty of Health, Deakin University, 221 Burwood Highway, Burwood, Victoria 3125, Australia; 2 Epworth HealthCare, Richmond, Victoria, Australia; 3 Cancer Council SA, Adelaide, South Australia, Australia; 4 Eastern Health, Department of Oncology, Box Hill, Victoria, Australia; 5 University of Newcastle, Newcastle, New South Wales, Australia; 6 Monash University, Melbourne, Victoria, Australia; 7 Barwon Health, Melbourne, Victoria, Australia; 8 Barwon South Western Regional Integrated Cancer Service, Geelong, Victoria, Australia

## Abstract

**Background:**

Carers provide extended and often unrecognized support to people with cancer. The aim of this study is to test the hypothesis that excessive carer burden is modifiable through a telephone outcall intervention that includes supportive care, information and referral to appropriate psycho-social services. Secondary aims include estimation of changes in psychological health and quality of life. The study will determine whether the intervention reduces unmet needs among patient dyads. A formal economic program will also be conducted.

**Methods/Design:**

This study is a single-blind, multi-centre, randomized controlled trial to determine the efficacy and cost-efficacy of a telephone outcall program among carers of newly diagnosed cancer patients. A total of 230 carer/patient dyads will be recruited into the study; following written consent, carers will be randomly allocated to either the outcall intervention program (n = 115) or to a minimal outcall / attention control service (n = 115). Carer assessments will occur at baseline, at one and six months post-intervention. The primary outcome is change in carer burden; the secondary outcomes are change in carer depression, quality of life, health literacy and unmet needs. The trial patients will be assessed at baseline and one month post-intervention to determine depression levels and unmet needs. The economic analysis will include perspectives of both the health care sector and broader society and comprise a cost-consequences analysis where all outcomes will be compared to costs.

**Discussion:**

This study will contribute to our understanding on the potential impact of a telephone outcall program on carer burden and provide new evidence on an approach for improving the wellbeing of carers.

**Trial registration:**

Australian New Zealand Clinical Trials Registry ACTRN: 12613000731796.

## Background

The replacement value of carers of cancer patients’ contribution in caring for family or friends is estimated at over $1 billion each year in the United States [1], with more recent estimates suggesting an average of $47,710 annually per carer [2]. Informal carers are important in the care of cancer patients, both economically and socially, with their role being complex and often lasting for years [3,4]. Carers often assume this role under sudden and extreme circumstances, with minimal preparation and limited guidance and support from the healthcare system [5,6]. Moreover, the role of the carer has shifted from promotion of gradual recovery to one of increased responsibility in the provision of more complex and prolonged care responsibilities [7,8]. The provision of care is often physically, emotionally, socially and financially demanding which results in the neglect of their own health needs [9,10]. A recent study showed that almost half of carers provided 21 to 40+ hours of unpaid care per week [5].

Carer burden is conceptualized as a demanding activity, or a negative reaction to activities related to caring for the patient [11,12]. These activities include providing practical day-to-day physical care and the resulting emotional reactions of the carer to the caregiving role, such as worry, depression, anxiety, frustration or fatigue [12].

Carers are at risk of excess psychological distress, sometimes even greater than that of the patient they are caring for [6,13,14]. Recent research highlighted that up to 70% of carers experienced depression [15,16] with 39% reporting significant depression compared to 23% of patients [17]. This has major implications for carers of more than 120,000 Australians estimated to be diagnosed each year [18]. In addition to depression, 17% to 47% of carers of people with cancer reported anxiety [4,19]. Psychological distress and depression are magnified if the carer is a spouse or adult child of the person with cancer and stage of disease [20,21].

Despite their substantial economic and social contribution**,** health services are not resourced to systematically support the practical and psychological needs of carers of patients undergoing active treatment for cancer. Consequently, carers are often inadequately prepared to manage the physical and emotional demands of caregiving [6,7,22]. There are few professional services or supportive care structures that accommodate or seek to address the needs of carers [23-26].

Despite the recognized burden, carer intervention research has been compromised by weak experimental designs that do not address carer needs [3,13]. Recent reviews of cancer carer studies found that carer interventions tended to address the patient’s care rather than dealing with carers’ unique needs [3,13,27,28]. Based on these findings, several areas were identified as being in need of further research; a) the identification of carers at higher risk of poor outcomes, so that interventions can be targeted to them; b) address socioeconomic differences; c) an economic evaluation, to understand the relationships between intervention delivery costs, health care utilization and intervention effectiveness, and d) the use of innovative technologies to deliver effective interventions.

The burden that carers experience is potentially modifiable by evidence-based, pragmatic interventions that meet carers’ needs. In the proposed study we will test the hypothesis that excessive carer burden is modifiable through a comprehensive low-cost intervention that includes supportive care and telephone support. The intervention involves taking established and successful telephone helpline services for patients and expanding them to proactively support carers of cancer patients. The intervention comprises:

a) A structured outcall program, of information and support to carers, delivered by Cancer Council Helpline nurses, that links carers to a range of community based supportive care services; and

b) Screening carers for distress and referring those with elevated levels to their general practitioner (GP) or other relevant services for follow-up to reduce their burden and improve their psychological health*.*

The Cancer Council Victoria (CCV) and Cancer Council SA (CCSA) Helplines are staffed by trained professionals, i.e. cancer nurses. Carers who have used this service in the past have reported high satisfaction, gains in knowledge, reduced distress, increased participation in decision making and increased sense of control [29]. At present, people must initiate contact with the Victorian and South Australian Cancer Council Helplines, a situation that applies to all existing Cancer Council telephone cancer services in Australia.

Access to a Cancer Council Helpline nurse will enable carers to discuss issues, independent of the patient, and link them to a range of community based supportive care services. Referral to psychological services will be responsive rather than reactive, will allow affected carers to receive appropriate support at an earlier and therefore more manageable stage.

### Pilot and previous relevant work

To inform the development of the intervention, we investigated the role of informal carers during cancer patients’ recovery from chemotherapy treatment. We found approximately 70% of cancer patients in our sample had an informal caregiver during some or all of the 5 days post chemotherapy. Carers highlighted significant gaps in the availability of information, communication with health professionals, financial assistance and practical support [30]. We investigated carer needs through six concept mapping workshops with carers, patients and health professionals. The results demonstrated significant gaps in information and support needs of carers during the treatment journey [31]. Our previous research involving a referral and telephone-based screening program of patients by cancer nurses on the Cancer Council Helpline also demonstrated the effectiveness of providing outcalls to cancer patients [32]. We also found that the telephone-based outcall program was feasible and acceptable for screening patients for distress, anxiety and depression [33]. Prior to implementation of the current study, we conducted a pilot study involving a sample of five carers to trial the procedures and outcall program with carers. The pilot study established the feasibility of the full-scale trial and acceptability of the outcall program to carers.

## Methods/Design

### Study design

The trial is a single blind, multi-centre, randomized, controlled trial to determine the efficacy and cost-efficacy of a telephone outcall program among carers of newly diagnosed cancer patients. A total of 230 carer/patient dyads will be recruited into the study, randomly allocated to either the outcall intervention group or an alternate minimal outcall service / attention control group (115 dyads in each group). Assessments will be at baseline, then at one and six months post-intervention. Deakin University Human Research Ethics Approval: 2012–083.

### Setting

**Figure 1:**
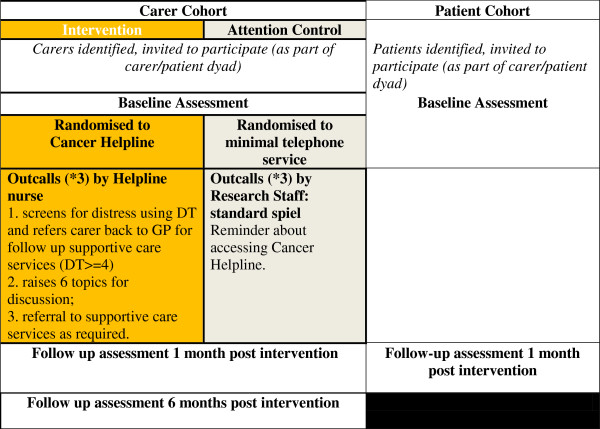
Study design involving carer and patient dyads.

The study will take place in three Victorian health services and one South Australian health service. These sites are typical public or private and urban or rural hospitals with socially diverse patient populations. An overview of the intervention and follow-up procedures is provided in Figure 1 below.

### Participants

A sample of 230 people newly diagnosed with cancer, *paired with their carers*, will be surveyed to determine potential changes in unmet needs over time. Participating carers / patient dyads will be allocated to the research arm via a computer-generated randomization scheme produced by the trial statistician.

#### Inclusion criteria

Adults, aged 18 years or more, who are carer / newly diagnosed cancer patient dyads, where the patient is attending cycles’ 2 to 5 of adjuvant chemotherapy or fractions’ 2 to 10 for radiotherapy treatment for cancer, at one of the four health services, who are able to complete English language questionnaires; cancer patients will be receiving treatment with curative intent.

#### Exclusion criterion

Cognitive dysfunction of either the cancer patient or the carer in the dyad. Experienced health service oncology nurses will determine cognitive dysfunction, defined as overt psychotic illness or dementia.

### Recruitment strategy

Carer/patient dyads will either be approached by an experienced clinical trials or research centre nurse, or by a researcher in the out-patient setting during presentation for adjuvant chemotherapy or radiotherapy.

Each dyad will be given a brief introduction to the study and initial consent will be sought for a researcher to contact them. Interested trial participants will be given a study pack (plain language statement, consent form and baseline questionnaire). Within approximately 48 hours carers / patients will be contacted by the project coordinator by phone and consent sought. Consenting carer/patient dyads will be asked to complete the consent form and baseline questionnaires and return them to Deakin University.

### Study entry procedures

Consenting carer/patient dyads will be randomized as described above. The names and telephone numbers of carers randomized to the intervention group will be emailed to the cancer nurse at the Cancer Council Helpline for subsequent follow-up. To ensure the integrity of the trial’s procedures, all study staff and clinical trial or research centre nurses will undergo specific training and quality assurance assessments throughout the study.

### Intervention program

To ensure a standardized intervention is applied, two Cancer Council Helpline nurses at each of the two Helplines will be specifically trained and will engage in ongoing service quality and consistency checks. Each will also serve as a backup to the other in case of illness. Seven Cancer Helpline outcall attempts over different times (e.g. 9 am-1 pm; 1 pm-5 pm; 5 pm-8 pm) across different week days over a two week period, will be undertaken. After seven failed attempts, a person will be declared ‘absent’ for a particular outcall. No further contact will be attempted until the next outcall is scheduled. All successful and failed attempts will be documented. Previous pilot work demonstrated that about 88% received the telephone outcall program under these conditions [32,33].

### Telephone outcall intervention

The standardized program, which is tailored to the individual, was developed through extensive consultation with Cancer Council Victoria Helpline nurses who regularly speak to carers of patients with all cancer types; representatives of Carers Victoria and carer workshops [31] and also incorporates evidence from the literature [3,11-13,16,27,28,34,35] to reflect the observed needs of a broad section of carers. Using a standardized protocol and checklist, the Helpline nurse will telephone participants 7 to 10 days post referral (Outcall One), four weeks later (Outcall Two), with the third outcall (Outcall Three) three months later. This time interval has been proven to be acceptable and effective in our previous research [32,33] and reflects periods of elevated stress during treatment and when people are approaching the survivorship phases of their condition. During each of the three outcalls, the Helpline nurse will administer the Distress Thermometer (DT) and will then raise six items for further discussion during which tailored information, support and specific carer resources (which have either been developed by Cancer Council Victoria or Cancer Council SA or are offered throughout the community) will be provided. Topics will be raised, the ensuing discussion will be then tailored to the participant’s needs and important aspects of the conversation will be documented.

The areas selected for discussion have been specifically chosen to achieve the intended outcomes of reduced carer burden, improved psychological health and quality of life and increased health literacy and ability to navigate the health system (as outlined in the introduction). The six areas that will be addressed are described in detail below.

1) **Psychological distress:** carer’s psychological/emotional and communication concerns; carers coping with their changed role and responsibilities; carer’s response to the patient’s cancer diagnosis and living with the side effects of treatment. Where the carer has cancer themselves, the impact on the patient’s diagnosis and own decision making will be discussed. The counsellor will aim to acknowledge and validate carer concerns.

2) **Health literacy:** carer’s understanding of treatment management issues and cancer terminology; the mechanisms for communication with specialist and how to navigate the health system and strategies for living with treatment side effects.

3) **Health:** diet / nutrition / exercise for both carer and patient*.*

4) **Family support**: partner/family issues and changes to intimate and sexual relationships.

5) **Financial impact:** cost of treatment, lost days from work / carer leave.

6) **Practical advice**: legal issues (Enduring Power of Attorney, Medical Power of Attorney, accessing Superannuation); advanced care planning, navigating the Centrelink (social security) system, respite care*.*

### Data collection

Carer screening for distress will be undertaken at each telephone outcall using the Distress Thermometer (DT). The DT is a two item screening tool for the detection of distress and/or depression and measures the impact of distress levels on daily life activity. Each ‘distress’ question is scored on a 0 to 10 scale, with scores ≥4 on the distress scale and scores ≥3 on the impact scale reflecting moderate levels of distress that have been found to warrant follow-up care [36]. This tool is reliable for community-based cancer helpline nurses to screen callers for distress [33,37]. Carers meeting cut-off scores on the DT will be referred back to either the established community-based Cancer Council psychological services (e.g. Cancer Council Counselling Service, Cancer Council Financial and Legal Assistance Program) or their GP for follow up and where required referral for psychological services through the GP Mental Health Care Plan (http://www.health.gov.au/internet/main/publishing.nsf/Content/pacd-gp-mental-health-care-pdf-qa). The GP Mental Health Care Plan provides a structured framework that supports referrals to clinical psychologists and allied mental health service providers. For those where depression or psychosocial issues are identified by the counsellor, participants will also receive referral to local health, welfare and support services as part of the tailored program.

### Control group: alternate minimal service

The alternate service or control group dyads will receive three telephone outcalls by members of the research team at the same times as the intervention group receives the outcall program. The purpose of these calls will be to remind participants about the availability of the Cancer Helpline. Participants who choose to contact the Cancer Helpline will not receive the outcall program but the usual support provided by cancer helpline nurses. Health services from which carers will be recruited have no systematic education or follow up programs for carers. Carers’ satisfaction with usual care provided by the hospitals, such as information and support, will also be collected at the health service level and by participant self-report at the one-month follow up.

### Data to be collected about carers: measures

Demographic characteristics to be recorded will include: carer age, gender, education, household size, postcode, health status, cultural background.

The primary outcome measure is a reduction in carer burden one month post-intervention. The Zarit Burden Interview (ZBI) is a 22 item, 5-point Likert scale (never = 0, nearly always = 4) used widely to assess carer burden [38]. The total burden is obtained by summing items to create a score from zero (lowest burden) to 88 (highest burden). The ZBI has been found to be psychometrically robust across carer groups [39]. In addition, carers will complete one sub-scale on the Caregiver Reaction Assessment (CRA) which comprises both positive and negative aspects of care-giving [34,35]. Each sub-scale is rated on a 5-point scale from “strongly disagree” to “strongly agree” [40]. For the purposes of this study, self-esteem will be included only; self-esteem is the positive reaction to care-giving and relates to the value that carers attribute to their role.

Symptoms of depression, a key secondary outcome, will be assessed using the Centre for Epidemiological Studies Depression Inventory (CES-D) [41]. This instrument has 20 items covering depressive symptoms and has been shown to have strong construct validity and concurrent validity when compared with clinical criteria and self-report assessments. It also has high internal consistency and acceptable test-retest reliability [42].

The Health Education Impact Questionnaire (heiQ) was designed to evaluate the intended benefits of a wide variety of self-management programs [43], is used in over 20 countries and has been adapted to the cancer setting [44]. It contains 40 questions across eight scales, each with high reliability. Two subscales, positive and active engagement in life and psychological distress, will measure the positive aspects of the caring role and the negative affective responses, respectively.

The Health Literacy Questionnaire (HLQ) will assess carers’ health literacy [45]. The HLQ consists of 44 items and was developed to measure a person’s capacity to seek, understand and use health information. The tool provides insight into client-practitioner interactions, guides program redevelopment and organizational responses to populations with low health literacy.

The Supportive Care Needs Survey—Partners and Caregivers (SCNS-P&C) is a 45 item tool which measures unmet needs of cancer carers across the illness trajectory. It comprises four domains: Health Care Service Needs, Psychological and Emotional Needs, Work and Social Needs, and Information Needs. It determines carers unmet needs and prioritizes health-care resources. The scale has been validated with carers of cancer patients [46,47].

The Assessment of Quality of Life (AQoL 8D) is a health-related quality of life utility measure. It generates quality-adjusted life-years used in economic evaluations and will assist with making judgements regarding value for money of the intervention compared with the control group. The AQoL 8D has been specifically developed for use in people with mental health problems or distress and is sensitive to change [http://www.aqol.com.au/].

The Resource Use Questionnaire will record health care resources used by carer participants. The questionnaire covers general health care services usage (self-reported), use of other welfare services, and effects on work force participation. The number of visits to health professionals during the survey period will be collected from all participants. The costs of consulting psychological professionals will be calculated using published prices for medical and allied health costs.

A satisfaction survey (intervention group) will be undertaken at one month post intervention to assess the acceptability of the intervention, the degree to which the intervention met their needs, including what elements of the intervention were or were not taken up, as well as information on carers’ access to, and use of, psychosocial services, access to, and use of, written information and referrals received from the nurse counsellors. In addition, satisfaction with the alternate service will be conducted (control group) to evaluate how the information provided by and referrals received from health professionals (e.g. doctors, nurses) met carers’ needs. Staff from both the Cancer Council Helplines and participating health services will be interviewed to determine the effect of the program on services, workloads, and resources, including the effect on waiting lists. Participants will be specifically asked for their views on the helpfulness of the calls and any concerns they may have had [32,33]. The number of referrals and take up of psychological services will be documented.

### People newly diagnosed with cancer: measures

Changes in self-management and health literacy will be ascertained using the heiQ and HLQ, depression levels using CES-D and unmet needs, using the Supportive Care Needs Survey (SCNS-SF34). The SCNS-SF34 is reliable and has demonstrated good content and construct validity for measuring global needs in cancer patients [48]. The tool was developed in Australia and is widely used within the Australian context [49,50].

### Sample size calculations

The primary outcome is change in burden by carers measured by the ZBI. Based on carer burden reported in several studies [51,52], we estimate carer burden in the sample at entry to our study to be moderate (mean ZBI = 18, SD = 13). With 180 carer participants (90 per treatment group) we will be able to detect a difference between the treatment groups of 6.3 units on the ZBI (for a two-sided alpha 5%, and 90% power. Allowing for 20% attrition, a sample of 230 dyads will be recruited into the study.

The secondary outcome is change in depression as measured by the CES-D*.* Based on previous research [15], to have sufficient power to detect a moderate difference of 5 points on the CES-D (SD = 10, for a two-sided alpha of 5% and 90% power) a total of 85 dyads in each group (a total of 170) is required. We will therefore have sufficient power to detect a moderate difference in our secondary outcome.

### Recruitment

Our previous work has shown that approximately 70% of cancer patients indicated they had a carer during the treatment phase of their condition [30]. Based on the number of new cases (~600/year; Health Services’ Information Services; 2011), if we assume that 1/3 of 600 new cases, each with a carer arrive for treatment at cycles’ 2 to 5 or fractions’ 2 to 10 across the four health services, a minimum of 16 carer /patient dyads will be recruited per month. Based on previous experience**,** we expect an 80% uptake [32,33] so we expect an overall recruitment phase of approximately 18 months to account for the greater challenge of recruiting the carer/patient dyad.

### Data analysis

### *Carer data*

An intention-to-treat (ITT) analysis will be performed [53]. Analysis of covariance will be used to assess differences in one and six month outcomes between groups, adjusting for baseline. Qualitative analysis techniques will be used to analyze data obtained from the telephone interviews.

### *Patient data*

An analysis of unmet needs will be ascertained at two time points, at baseline and one month post intervention period, among participants with cancer.

### Economic analysis

This study will be the first carer intervention to be subject to a formal economic evaluation and is in keeping with the recommendations of Northouse and colleagues [5] in their meta-analysis of interventions with family carers of cancer patients. This evaluation will comprise a cost-consequences analysis where incremental costs of the intervention will be compared with the outcomes included in the study. This means that a series of cost-effectiveness ratios will be determined rather than just one, such an approach has been shown to be useful for decision-makers. Inclusion of the AQoL-8D will also enable a cost-utility analysis to be undertaken, thereby allowing practical judgments to be made regarding value for money credentials of the intervention. The economic analysis will be primarily from the perspective of the health care sector though and a secondary analysis from the broader societal perspective will also be undertaken. The evaluation will first measure and value any change to the use of health care resources over the period of the study between the two arms of the trial (intervention and control) and then compare any additional costs to the additional outcomes achieved. Resource use over time will be accessed via the resource use questionnaire. Micro costing techniques will be used to determine the costs of the intervention. Standardized economic evaluation techniques will be used including incremental analysis of mean differences and bootstrapping to determine confidence intervals.

## Discussion

With the ageing of the population, the number of new cases of cancer diagnosed in Australia is expected to reach 150,000 in 2020 [54]. We therefore expect to see an increase in the number of people living with the disease [18,54] and a concomitant increase in the number of carers who are caring for a person with cancer. This study will investigate the impact of a novel intervention on health outcomes among carers. If this research demonstrates the PROTECT intervention is effective and cost effective, health services will have a new resource which is a strong interface between clinical care and community based supportive care. Moreover, it has the potential to reduce health inequalities as the use of a telephone service means that access to information and counselling is not restricted by geographic (urban, rural) boundaries, nor limited by transportation barriers. If the intervention is found to be effective, it has the potential to be established in cancer helplines across all states, servicing public and private as well as metropolitan, rural and regional health services throughout Australia. Given the contribution carers make to Australian society, and the personal and financial burden that these individuals carry, an effective intervention that facilitates carers’ important contribution will be timely.

## Competing interests

The authors declare that they have no competing interests.

## Authors’ contributions

PML conceived the idea with input from RO and MB; CM designed the economic component of the study. PML, RO, MB, CM, S McG, LH, KG, JC, DA and MW provided substantial input into the development of the protocol. PML and LH drafted the manuscript with contributions from RO, MB, CM, S McG, KG, JC, DA and MW. Each of the co-authors are on the steering committee, oversee implementation of the study and data collection. Each of the authors contributed to, read and approved, the final manuscript.

## Pre-publication history

The pre-publication history for this paper can be accessed here:

http://www.biomedcentral.com/1472-6963/14/5/prepub
